# Geospatial Analysis of Orthopedic Workforce Distribution Across Georgia

**DOI:** 10.7759/cureus.95588

**Published:** 2025-10-28

**Authors:** Dev Shah, William E Long, Shervin Eskandari, Anna Bozzone, Stephen A Parada

**Affiliations:** 1 Orthopaedic Surgery, Augusta University Medical College of Georgia, Augusta, USA

**Keywords:** access to care, georgia, orthopaedics, rural healthcare access, workforce distribution

## Abstract

Introduction: Healthcare disparities between rural and urban populations have been well documented; however, orthopedic surgery-specific care remains under-characterized in rural communities. The purpose of this study was to assess rural-urban disparities in access to orthopedic care in Georgia.

Study type: This is a cross-sectional study.

Methods: The Georgia Composite Medical Board database was utilized to identify practicing orthopedic surgeons and their location of practice. County-level demographic data, including population and median household income, were collected from the 2020 United States Census. Counties were stratified using the 2023 Rural-Urban Continuum Codes, and they were sorted into metropolitan (categories one to three) and non-metropolitan counties (categories four to nine). Metrics calculated included orthopedic surgeon density per 100,000 residents, surgeon density per 100 square miles, average travel distance from the county centroid to the nearest orthopedic practice, and income correlation per county.

Results: A total of 838 orthopedic surgeons were identified across Georgia’s 159 counties. Metropolitan counties had significantly higher provider density than non-metropolitan counterparts, both per 100,000 residents (4.81 vs. 2.56, p = 0.009) and per 100 square miles (3.07 vs. 0.22, p < 0.001). Rural counties also experienced a greater travel burden to the nearest orthopedic provider (15.85 vs. 8.19 miles, p < 0.001), and fewer counties had at least one orthopedist (34.9% rural vs. 54.1% urban). Correlation analysis revealed that lower income was associated with both greater travel burden (ρ = -0.47, p < 0.001) and lower provider density per capita (ρ = 0.34, p < 0.001).

Conclusion: This study highlights a significant gap in access to orthopedic care between rural and urban counties in Georgia, with individuals in rural counties having limited access to orthopedic care due to longer travel distances and lower surgeon density.

## Introduction

Geographic disparities in healthcare access remain an issue in the United States, impacting rural populations who have reduced access to medical care compared to urban areas [1]. This disparity is especially prominent in subspecialty care, such as orthopedic surgery [2,3]. Furthermore, rural orthopedic surgeons tend to be older than their urban counterparts, with nearly 50% of rural orthopedic providers being aged 55 or older, raising concerns about upcoming workforce stability and the sustainability of orthopedic access in these regions [4]. Orthopedic surgery provides patient care by addressing common conditions such as musculoskeletal injuries, degenerative joint diseases, and trauma care, conditions that require access to physicians for effective care. However, the skewed distribution of orthopedic surgeons toward urban areas indicates accessibility concerns for rural populations [2-4].

Georgia's population distribution offers a unique situation to study these disparities, as 57% of Georgia’s population resides in the metropolitan Atlanta area [5]. Additionally, approximately 21% of Georgia’s population of 10.7 million residents live in rural areas, signifying the need to address problems that these populations may be facing [5]. Restrictions in accessing healthcare for residents in rural counties are amplified by a lower average socioeconomic status, less developed transportation infrastructures, and reduced accessibility to healthcare resources. Recent reports from the Health Resources and Services Administration (HRSA) have projected a worsening of the shortage of orthopedic surgeons, which is expected to disproportionately affect rural areas [6]. This is occurring while the demand for orthopedic services is expected to rise with the aging population, specifically pertaining to total joint arthroplasty and fracture care [7].

Prior studies examining geographic disparities have evaluated shortages in numerous specialties [8]. However, analysis of these geographic gaps specific to orthopedic surgery in Georgia has not yet been explored. Given the value of early and regular orthopedic management in improving quality of life and functional outcomes, quantifying these disparities is essential. The purpose of this study was to analyze orthopedic care availability and accessibility throughout Georgia to identify any significant geographic or accessibility disparities. We hypothesize that rural counties in Georgia will face significantly lower access to orthopedic care compared to urban counties, and that this disparity will correlate with economic disadvantage, limitations of healthcare infrastructure, and population density.

For the purpose of this study, the term orthopedic workforce was defined as board-certified or board-eligible orthopedic surgeons licensed and actively practicing in Georgia. Access to care was defined as the physical availability and geographic proximity of these providers to the population, measured through surgeon density and travel distance.

## Materials and methods

To analyze the distribution of orthopedic surgeons across the state of Georgia, a cross-sectional study was performed utilizing the Georgia Composite Medical Board data [9]. The Georgia Composite Medical Board tracks all practicing healthcare professionals in Georgia. This data was used to extract a list of all orthopedic surgeons practicing in Georgia as well as their primary office locations. Each orthopedic surgeon was counted according to their primary practice address listed in the Georgia Composite Medical Board database. Surgeons with multiple practice sites were assigned to their primary office location. As a result, surgeons who practice across multiple counties may not be fully represented, potentially underestimating access in some regions. Travel burden was calculated as the straight-line (Euclidean) distance from each county's centroid to the nearest orthopedic practice. While this method approximates geographic access, it does not account for actual driving routes or travel conditions and may underestimate real-world travel distances.

The 2020 United States Census was utilized to obtain data on Georgia's 159 counties. Data on each county’s population, land area, and median household income were recorded. To further classify counties and categorize them, the 2023 Rural-Urban Continuum Codes were used [10]. The 2023 Rural-Urban Continuum Codes are separated into nine codes (one to nine), with three (one to three) being metropolitan areas and six (four to nine) being non-metropolitan. The metropolitan counties are sorted by population, with code one being counties with a metro population of one million or more, code two being counties with metro populations between 250,000 and one million, and code three being counties with fewer than 250,000 residents. Non-metropolitan counties (codes four through nine) are classified based on population size and proximity to metropolitan areas. These include (code four) urban counties bordering a metro area with 20,000 or more residents, (code five) urban counties not bordering a metro area with 20,000 or more residents, (code six) urban counties adjacent to a metro area with populations between 5,000 and 20,000, (code seven) urban counties not adjacent to a metro area with a population between 5,000 and 20,000, (code eight) counties near a metro area with a population below 5,000, and (code nine) those with fewer than 5,000 residents that are not near any metro area.

For each county, the total number of surgeons and the number of orthopedic surgeons per 100,000 residents were determined. To calculate geographic spread, density per 100 square miles was also obtained. To identify county-specific travel burden, the straight-line (Euclidean) distance from the geographic centroid of each county to the nearest orthopedic surgery practice was measured. Mann-Whitney U and Spearman correlation tests were used for group comparisons and associations. All data analysis was conducted utilizing SPSS v30.0 (IBM Corp., Armonk, NY, US).

## Results

Analysis of all 159 counties in Georgia revealed significant disparities in orthopedic care access between metropolitan and non-metropolitan regions. Across the state, a total of 838 orthopedic surgeons were identified. Table 1 presents county-level data stratified by the rural-urban classification code.

**Table 1:** Data for Georgia Counties by Rural-Urban Classification *Values are statistically significant (p < 0.01)

Rural-urban classification code	Counties	Total population	Rural population	Total % rural	Total square miles	Total no. of orthopedists	Average orthopedists/100k*	Average orthopedists/100 square miles*	Average travel burden (miles)*
1	29	6,104,803	737,128	12.10%	9,114	467	4.83 (4.6)	4.15 (6.82)	5.67 (6.62)
2	17	1,242,810	287,299	23.10%	6,186	142	4.35 (7.55)	2.87 (6.02)	11.11 (8.67)
3	28	1,577,355	542,155	34.40%	10,222	154	5.07 (7.45)	2.08 (4.05)	9.03 (6.90)
4	9	520,764	259,398	49.80%	4,337	30	6.07 (5.73)	0.72 (0.68)	4.48 (5.46)
5	2	77,595	28,405	36.60%	1,160	9	11.23 (8.07)	1.45 (1.74)	1.77 (0.37)
6	16	426,970	255,040	59.70%	5,730	18	3.6 (4.8)	0.28 (0.35)	10.68 (9.37)
7	8	195,225	118,359	60.60%	3,749	9	4.22 (3.88)	0.22 (0.23)	13.45 (11.64)
8	31	391,092	384,610	98.30%	11,546	8	1.4 (3.12)	0.08 (0.18)	21.21 (12.04)
9	19	175,294	165,528	94.40%	5,671	1	0.31 (1.36)	0.01 (0.06)	19.36 (10.88)
Total	159	10,711,908	2,777,922	25.90%	57,716	838	3.61 (5.48)	1.55 (4.15)	12.29 (10.83)

Metropolitan counties (n = 74) accounted for most orthopedic providers (763 of 838 total), while non-metropolitan counties (n = 85) had only 75. The average number of orthopedic surgeons per county was significantly higher in metropolitan areas (10.31 vs. 0.88, p < 0.001). A total of 34.9% of rural counties had at least one orthopedic surgeon, compared to 54.1% of urban counties. The top five counties with the highest orthopedic density were all metropolitan. Orthopedic density per 100,000 residents was significantly greater in urban counties (4.81 vs. 2.56, p = 0.009), as was density per 100 square miles (3.07 vs. 0.22, p < 0.001).

Counties classified under codes eight and nine (representing the most rural areas) had the lowest densities, averaging just 1.4 and 0.31 providers per 100,000 population, respectively, and the lowest presence per square mile (0.08 and 0.01). Table 2 summarizes the differences between metropolitan and non-metropolitan counties across socioeconomic factors, including household income and orthopedist density.

**Table 2:** Orthopedic Access in Metropolitan and Non-metropolitan Counties. Data Presented as Mean (Standard Deviation) Where Applicable

	Metropolitan	Non-metropolitan	p-value
Number of counties	74	85	–
Total population	8,924,968	1,786,940	–
Total rural census	1,566,582	1,211,340	–
Total % rural	17.6%	67.8%	–
Total square miles	25,522	32,194	–
Total orthopedists	763	75	–
Average square miles	344.90 (130.98)	378.75 (166.84)	0.442
Average % rural	54.40% (36.10)	80.11% (23.20)	<0.001
Average median household income	$70,153.81 ($18,908.06)	$52,072.22 ($10,011.87)	<0.001
Average number of orthopedists	10.31 (23.39)	0.88 (1.72)	<0.001
Average orthopedists/100k	4.81 (6.42)	2.56 (4.28)	0.009
Average orthopedists/100 square miles	3.07 (5.72)	0.22 (0.45)	<0.001
Average travel burden (miles)	8.19 (7.46)	15.85 (12.03)	<0.001

**Figure 1:**
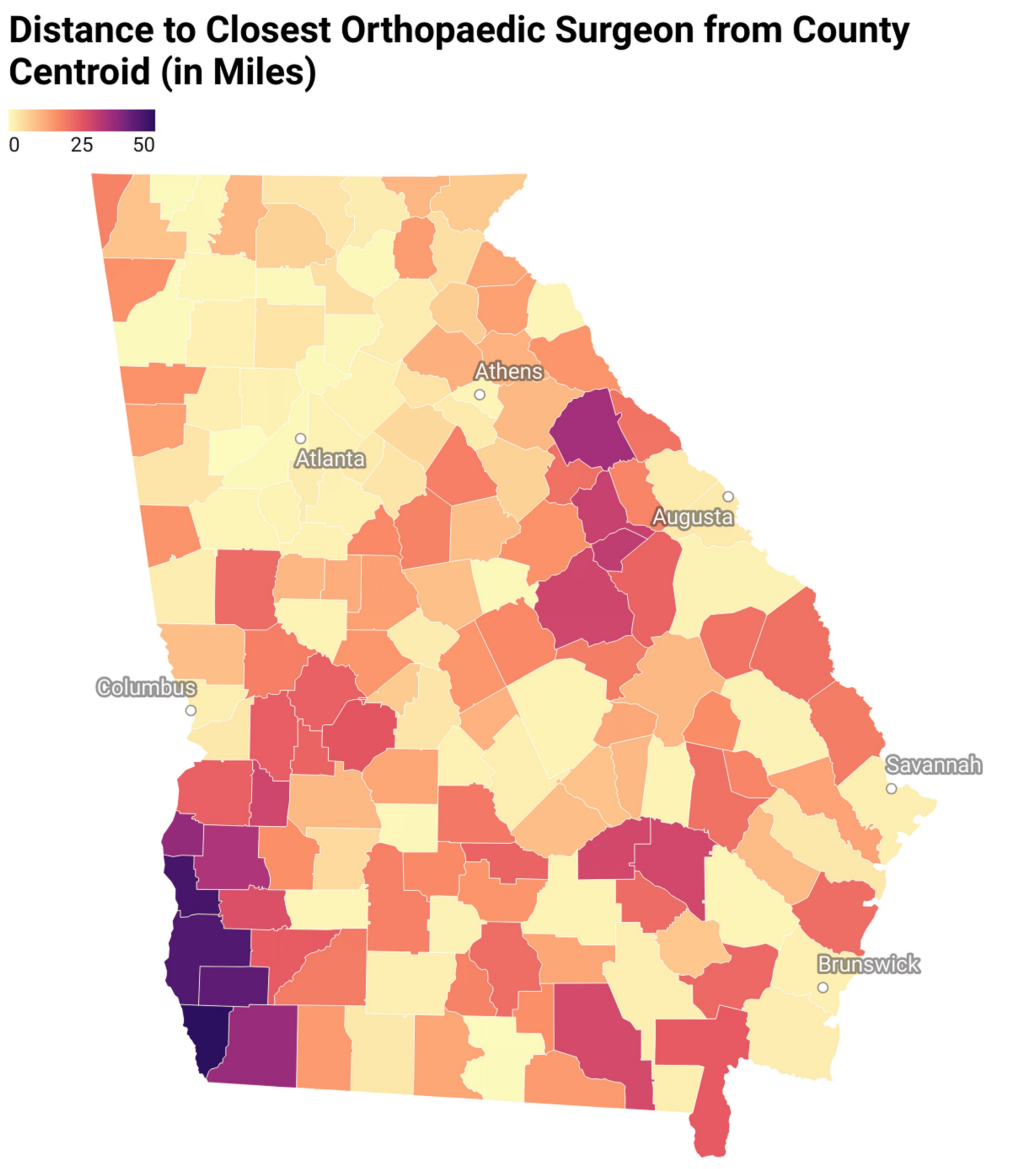
Distance to Closest Orthopedic Surgeon From County Centroid (in Miles) Figure created by the authors using Datawrapper

Residents in rural counties faced significantly longer travel distances to orthopedic care. The average linear travel distance from the county centroid to the nearest provider was 15.85 miles in non-metropolitan counties compared to 8.19 miles in metropolitan counties (p < 0.001). Counties falling under code eight had the highest average travel burden (21.21 miles), with some exceeding 30 miles. Rural counties also demonstrated lower socioeconomic standing. The average median household income in rural counties was $52,072, compared to $70,154 in urban counties (p < 0.001). Spearman correlation tests revealed a significant negative association between household income and travel burden (ρ = -0.47, p < 0.001), indicating that lower-income counties were farther from orthopedic care. A positive correlation was observed between income and surgeon density per 100,000 residents (ρ = 0.34, p < 0.001), suggesting that higher income areas have better access to orthopedic care. Figure 1 displays the distance from the centroid of each Georgia county to the nearest orthopedic surgeon, highlighting the longer travel distances those in rural counties face.

Among the 85 non-metropolitan counties, only 16 had at least one orthopedic surgeon. Several counties had no orthopedic providers within county lines and required travel of over 20 miles to their closest provider. These findings highlight a clear skew of access to orthopedic care with potential implications for delays in treatment, poorer outcomes, and overburdened urban providers.

## Discussion

This study highlights the significant rural-urban divide in orthopedic surgical access across the state of Georgia. Rural counties face a multifactorial burden; not only are there fewer orthopedic surgeons available, but these areas also show lower population density, weaker infrastructure, and reduced economic means to bridge these gaps. Factors such as income, infrastructure, and population density are closely interrelated, making it difficult to attribute disparities in orthopedic access to any single variable. These results are consistent with national patterns documented across other specialties but fill a crucial gap in our understanding of orthopedic workforce distribution [8].

Similar disparities have been documented in prior state-level and national analyses of the orthopedic workforce. Hughes et al. examined the orthopedic surgeon distribution in Kansas and found a similar pattern, with higher concentrations in metropolitan counties and markedly lower densities in rural areas [2]. At a national level, Fu et al. reported comparable rural-urban discrepancies, noting that rural counties not only had fewer providers but also an aging surgeon population, raising concerns about the long-term workforce sustainability [4]. Broader evaluations of physicians across other specialties have echoed these findings, with rural areas consistently facing reduced access to specialty care across multiple specialties [1,8]. Our results from Georgia align with these other studies and reinforce the importance of addressing this orthopedic maldistribution.

One possible cause of the rural-urban disparities in orthopedic access could be explained by the high dependence of rural patients on Medicaid [11]. Patients in rural communities may not have the insurance coverage necessary to support full-time subspecialists, further disincentivizing development of practices in these regions [11]. This mirrors patterns observed in other specialties, such as otolaryngology and general surgery, but the procedural and required follow-up appointments unique to orthopedics may exacerbate the impact of access disparities [12,13].

The relationship between income and access underscores the socioeconomic dimension of this problem [12,13]. Counties that had higher household incomes not only showed shorter distances to orthopedic care but also higher densities of orthopedic surgeons. This suggests that economic factors may drive the distribution of the orthopedic workforce. One way to help alleviate this disparity would be to potentially utilize these findings when designing healthcare reform programs designed to redistribute the specialist workforce to increase accessibility.

Strategies that can help alleviate this disparity may include expanding rural-focused graduate medical education programs, offering targeted student loan forgiveness, and progressing accessibility using telemedicine in orthopedics to extend access into underserved regions. Additionally, team healthcare models involving physician assistants and nurse practitioners could serve as a scalable approach to bridging care gaps in these regions [14]. Even with the rise in the use of telemedicine, orthopedic care is heavily reliant on physical examinations, imaging, and operative care, limiting the extent to which virtual models can fill these gaps. Further strategies to address this disparity may include expanding rural-focused graduate medical education programs, especially orthopedic surgery residencies that include rural exposure or longitudinal rural involvement. Mobile clinics and outreach programs have been piloted in other surgical specialties with success, demonstrating that this solution may help acutely aid orthopedic care gaps in underserved counties [15]. While the present study is limited by its cross-sectional design and does not account for multiple practice locations (such as those working across multiple counties), the geographic span of this study and analysis of data provide a strong foundation and call for an improvement in orthopedics access to care. Future studies exploring these disparities could be focused on evaluating longitudinal trends in the workforce and exploring how access disparities are linked to patient outcomes.

## Conclusions

This study highlights large disparities in access to orthopedic surgical care between rural and urban areas of Georgia. With significantly fewer providers per capita, greater travel distances, and lower average household incomes, rural communities face numerous structural barriers in receiving timely orthopedic care. Without reliable access to orthopedic care, rural residents face greater risks of delayed medical treatment, which could lead to worsening conditions, prolonged disability, and a reduced quality of life. Addressing these disparities will require significant changes in medical education initiatives, policy modifications, and infrastructure developments to ensure equitable access to surgical care for all Georgians, regardless of geography.
